# Experimental study on the backdraft phenomenon of solid fuel

**DOI:** 10.1371/journal.pone.0255572

**Published:** 2021-08-02

**Authors:** Jianlong Zhao, Yanfeng Li, Junmei Li, Youbo Huang, Jinxiang Wu

**Affiliations:** 1 Beijing Key Laboratory of Green Built Environment and Energy Efficient Technology, Beijing University of Technology, Beijing, China; 2 College of Safety Engineering, Chongqing University of Science & Technology, Chongqing, China; 3 School of Energy and Environmental Engineering, Hebei University of Technology, Tianjin, China; China University of Mining and Technology, CHINA

## Abstract

In this study, a series of small-scale experiments were conducted to investigate the backdraft phenomenon in a compartment (1.1 × 0.4 × 0.75 m) with woodblocks as fuel. This research focused on the effects of compartment window woodblock areas on backdraft time, with a video recording of the experimental phenomena. Thermocouples and a gas analyzer were used to measure the temperature and the concentration of gas components in the compartment, respectively. There was no additional heat source or ignition source pre-set in the compartment at the beginning of the experiments; the experimental processes only depended on the heat released from the burning or smoldering of woodblocks. When compartment ventilation improved, smoldering of woodblocks became intense, and backdraft occurred. The results show that backdraft time is shorten with increases of compartment window and woodblock areas, and opening the upper window of a compartment could avoid the backdraft phenomenon. The results help to understand the backdraft phenomenon of solid fuel and, more importantly, could help firefighters adopt reasonable fire-fighting strategies for restraining backdraft occurrence.

## 1. Introduction

In recent decades, many backdraft fires have occurred [1–4]. These backdraft fires have led to huge casualties and large property losses; therefore, backdraft maybe one of the most dangerous special fire behaviors [5]. Moreover, because backdraft threatens the safety of firefighters, it has also been described as a killer of firefighters [6].

To avoid casualties and property losses caused by backdraft, we need to understand the phenomenon of backdraft. Generally speaking, backdraft is a special fire behavior due to under-ventilated conditions in a compartment [7]. The IFE [8] and NFPA [9] provide a detailed description of the backdraft phenomenon.

Fleischmann et al. [10–15] first systematically studied the backdraft phenomenon. They conducted preliminary studies on many aspects of the backdraft phenomenon such as gravity current, explosive fireball, measured ignition delay, deflagration travel times, temperature and pressure rise in a compartment during backdraft, and the mass fraction of unburned hydrocarbon species to determine the characteristics of its development. Their studies suggested that backdraft occurred when the unburned hydrocarbon concentration in a compartment became greater than 10%, and that the intensity of backdraft could be enhanced by the concentration of the fuel.

Gottuk et al. [16] conducted large-scale experiments to explore the initial critical conditions and suppression methods of backdraft. They suggested that the critical initial fuel mass fraction that determines the occurrence of backdraft occur 16%. In addition, reducing the fuel mass fraction by spraying water mist could avoid the occurrence of backdraft. Tsai et al. [17] also conducted three large-scale backdraft experiments using solid fuel. During the experiments, the development and phenomenon of backdraft were similar to the actual situation. The results showed that backdraft caused two temperature peaks. Hu et al. [18] and Shi et al. [19] both conducted full-scale experiments to investigate the temperature distribution in a long-narrow space.

Because large-scale backdraft experiments are expensive, some studies have been conducted as reduced-scale experiments.

Weng et al. [20–22] carried out reduced-scale experiments to study the backdraft phenomenon. According to their results, they established a simplified mathematical model of backdraft which showed the dependence between control variables of the system and fire condition. They also found that, when backdraft occurred, the mass fraction of hydrocarbon was a critical value that depended on the opening geometry of a compartment.

Gong et al. [23] established a dynamic model based on the backdraft phenomenon of liquid fuel, which could be used to describe the temperature of a hot smoker layer. Wu et al. [24] carried out reduced-scale experiments and investigated the effects of ventilation conditions, ignition locations, and mass fluxes of gas fuel on backdraft. Their results suggested that ventilation conditions were the most critical factor which determined the occurrence of backdraft.

Chen et al. [25] conducted reduced-scale experiments to study the backdraft phenomenon of solid fuel. According to the experimental results, they established a theoretical model and deduced the critical condition of backdraft. Wu et al. [26, 27] conducted reduced-scale experiments to study temperature conditions and critical factors of backdraft. Their results indicated that before the opening of a compartment, maximum temperature and the CO/CO2 ratio in the compartment were crucial factors that determined whether backdraft occurred or not. Tang et al. [28] and Mei et al. [29] conducted model-scale experiments to investigate the evolution of the maximum temperature and thickness of a smoke layer in a tunnel with ceiling extraction.

In recent years, studies have investigated the backdraft phenomenon using numerical simulations. Hu et al. [30] analyzed the longitudinal profile of CO concentration and temperature rise on a tunnel fire. Ko et al. [31] investigated the effect of ignition locations and times on the backdraft behavior in a compartment using Fire Dynamics Simulator (FDS) code. Myilsamy et al. [32, 33] studied the occurrence of backdraft in a compartment filled with high-temperature methane fuel, and the effect of the compartment opening geometry on backdraft by FDS code; the results proved this code could be used to investigate the backdraft phenomenon. Ha and Oh [34, 35] conducted a Large Eddy Simulation (LES) and successfully performed the backdraft phenomenon, and then investigated the effect of opening geometries on backdraft. Ashok et al. [36] used FDS code to study the effect of the gravity constant on backdraft and found that between them there was a strongly nonlinear relationship. Król et al. [37] used Ansys Fluent to study the backdraft phenomenon and revealed the characteristics of backdraft and the occurrence of the gravity current.

The above-mentioned studies suggest that after a combustible gas has filled up a ventilated confined compartment, when the compartment ventilation improves, fresh air flows in, mixes with this combustible gas, and finally forms a mixture. Once the mixture reaches the flammability limits, a backdraft occurs. The combustible gas can be gas fuel, vapors generated from the evaporation of liquid fuel, or pyrolysis production of solid fuel.

However, studies on the backdraft phenomenon have mostly used gas fuel and rarely used solid fuel. In addition, previous studies have pre-set the ignition position, used an additional heat source, or improved the ventilation conditions when flames still existed in the compartment. These operations are different from the actual process of a compartment fire. During an actual compartment fire, the ignition location is random, and heat is released by combustion but an additional heat source. More importantly, ventilation conditions are not improved when flames still exist in the enclosed compartment, preventing fresh air flow into the compartment and avoiding the intense of fire.

On the basis of actual backdraft fires, we reproduced the backdraft phenomenon through small-scale experiments. There was no additional heat source or ignition source in the compartment. Therefore, the experiments were promoted by solid fuel burning or smoldering. In addition, when the compartment window opened, flames on the solid fuel surface were distinguished. In this study, we investigated the influences of compartment window area and solid fuel area on backdraft time. The results should help to understand the backdraft phenomenon of solid fuel. More importantly, they should help firefighters adopt reasonable fire-fighting strategies for restraining backdraft occurrence.

## 2. Experimental set-up

**Fig 1:**
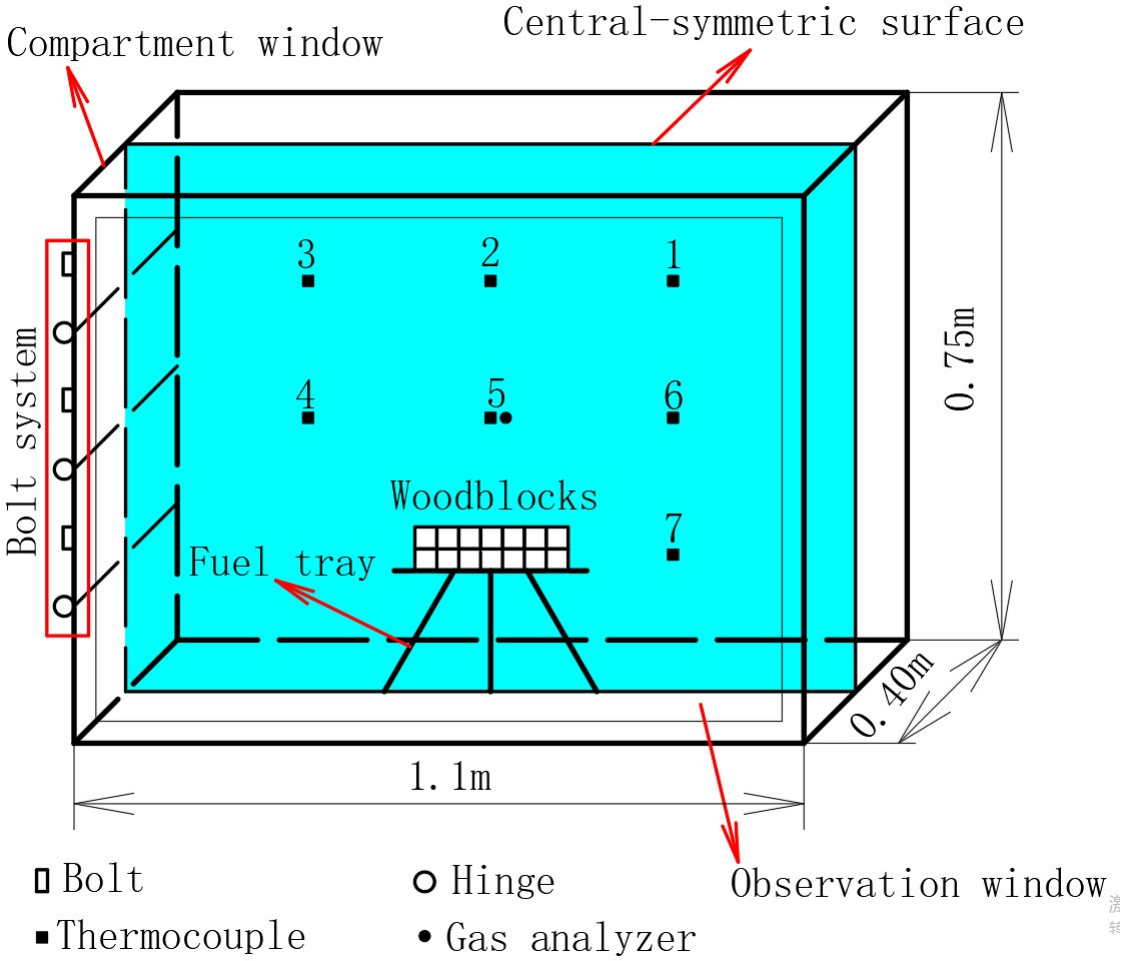
Model of the experimental compartment.

Fig 1 is the schematic configuration of the compartment that was used in our previous study [24]. The compartment is 1.1 × 0.4 × 0.75 m (L × W × H), made of 2.5-mm-thick stainless steel, welded together to ensure strength. The interior volume of the compartment is 0.33m^3^ and denoted as V. There is an observation window in one of the long walls of the compartment. The observation window is made of quartz glass that is 1.08 × 0.72 m (L × H). During the experiments, solid fuel was placed on a fuel tray at the bottom and in the middle of the compartment.

The horizontal and vertical trisect of the central-symmetry surface were the measuring point positions. There were seven thermocouples in the compartment, denoted as T1~T7, consecutively. There was only one thermocouple set at the bottom of the compartment. This because preliminary experiments have suggested that temperature varies on both sides of the fuel tray were similarly. In addition, the closer to the bottom of the compartment, the smaller the temperature difference between the two sides. The gas analyzer was set at the 5th position.

**Fig 2:**
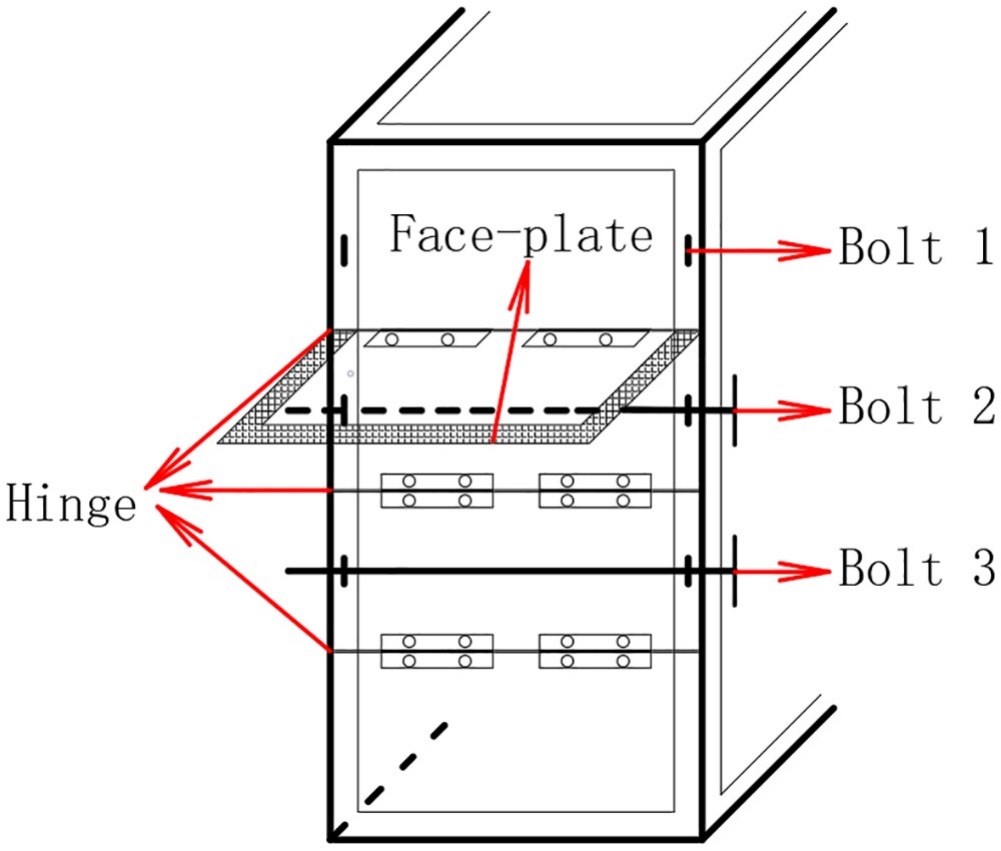
Sketch of the bolt system of a compartment window.

Fig 2. presents the bolt system of the compartment, which included three hinges, three bolts, and three face-plates. By removing bolts and opening the face-plate, the compartment window area could be changed to three different values, denoted as S_1_. By removing Bolt 1, one face-plats was opened and the compartment window area was 0.075 m^2^. By removing Bolt 1 and 2, two face-plates were opened, and the compartment window area was 0.15 m^2^. By removing Bolts 1, 2, and 3, three face-plates were opened and the compartment window area was 0.225 m^2^.

The solid fuel used in this research was woodblocks formed by crushing wood chips. The shape of each woodblock was cubic (side length was 3.5 cm). The woodblocks had three different place forms along with the direction of length and width of the compartment. The number of woodblocks corresponding to these forms was 4 × 4, 5 × 4, and 6 × 4, respectively. There were almost no gaps between woodblocks which were tightly placed. Therefore, the woodblocks’ upper surface areas of the above three-place forms were approximately 0.0196, 0.0245, and 0.0294 m^2^, respectively, and denoted as S_2_. To ensure the total number of woodblocks during experiments and to avoid too many woodblocks left after the experiments finished. There were two layers of woodblocks placed on the fuel tray, approximately 7 cm high.

At the beginning of the experiments, the compartment window area was 0.15 m^2^. The woodblocks were placed on the fuel tray, and the fuel tray was placed at the bottom and middle of the compartment. Then, alcohol was sprayed on the upper surface of the woodblocks and rapidly ignited. After 360 seconds, the compartment window was closed, the flames on the woodblocks were extinguish due to oxygen concentration reduction, and then the woodblocks converted to smoldering. After 120 seconds of the flames’ extinction, the compartment window was opened. Then, fresh air flowed into the compartment, the smoldering of woodblocks became more and more intense, and backdraft finally occurred. The backdraft represents the time from opening the compartment window to the time a flame appears on the woodblock’s surface, denoted as t. The experimental settings are shown in the Table 1.

**Table 1:** Experimental settings.

Test No.	The upper surface area of wood blocks S_2_ (m^2^)	The window area of the compartment S_1_(m^2^)	The backdraft time t(s)
1	0.0196	0.075	732
2		0.15	392
3		0.225	72
4	0.0245	0.075	560
5		0.15	350
6		0.225	50
7	0.0294	0.075	502
8		0.15	342
9		0.225	44

## 3. Results and discussion

### 3.1. Experimental phenomena

The following is a detailed description of the experimental phenomena, using Test 8 as an example.

**Fig 3:**
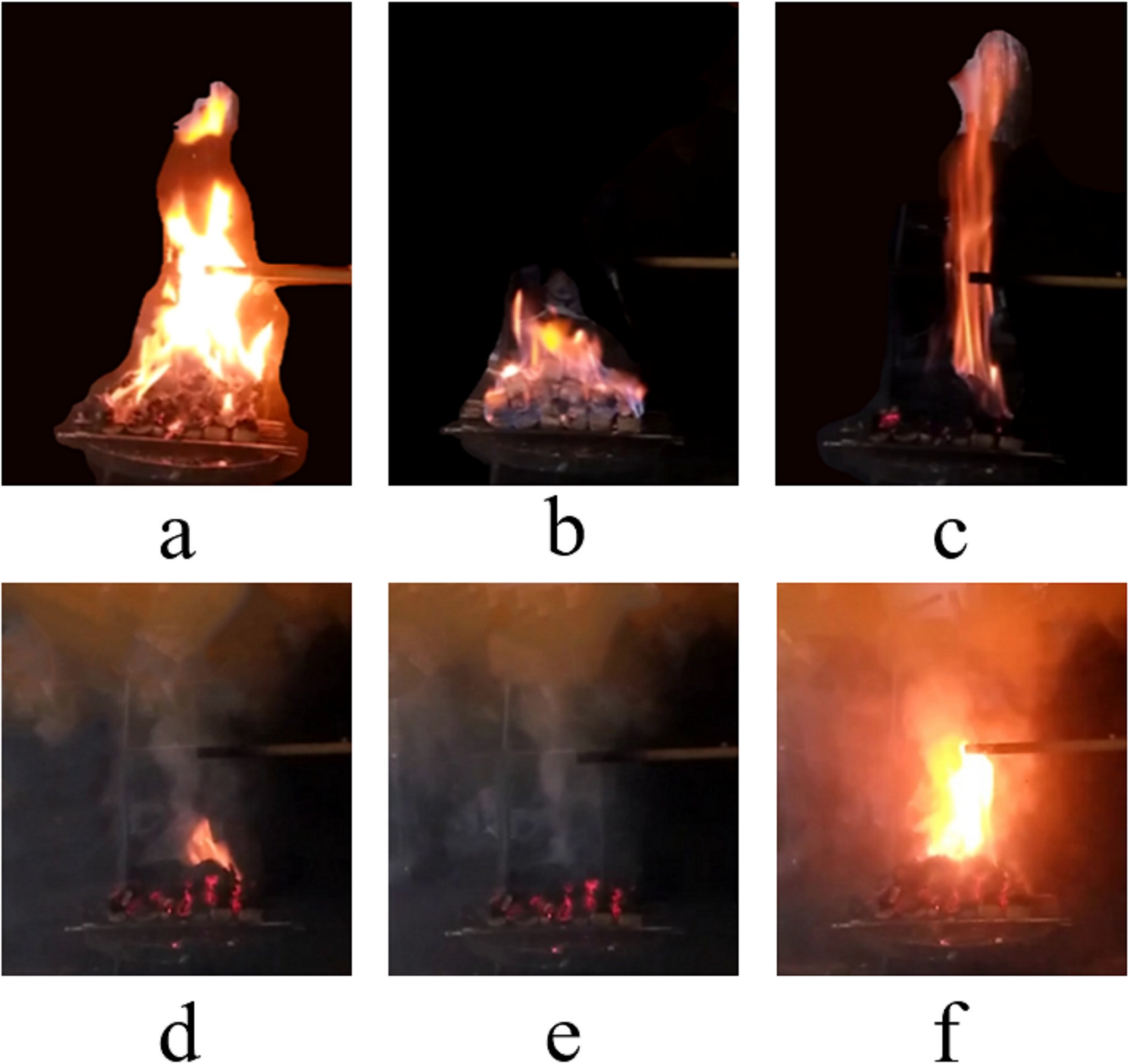
The flame on the surface of wood blocks of Test 8: (a) bright light blue flame; (b) bright yellow flame; (c) vertical flame; (d) intense smoldering and appearance of high temperature area; (e) appearance of flame (f) rapid covering of flame on the surface of the wood blocks.

At the time of ignition, first, the alcohol sprayed on the surface of the woodblocks started burning, and the flame was a bright light blue (Fig 3a). When the heat released by the burning alcohol heated the upper surface of the woodblocks to the ignition temperature, the woodblocks also started burning. Then, the light blue flame gradually faded, and some yellow flames begin to appear. After the alcohol was burned out, the woodblocks burn steadily, and therefore the flames became a bright yellow (Fig 3b).

After the compartment window was closed, the woodblocks continued to burning because the oxygen concentration in the compartment can sustain burning for a short time period. However, as the oxygen concentration decreases, finally, to below the flammability limits, the flame gradually darkens and was eventually extinguished. During this period, because the flame was not disturbed by the external environment, it remains vertical (Fig 3c). Finally, the flame detached from the upper surface of the woodblocks, and then extinguished in the process of upward lifting.

When the compartment window was opened, smoke flowed out from the compartment, and fresh air flowed into the compartment. The oxygen concentration in the compartment gradually increased with an exchange of smoke and fresh air. Then, smoldering of the woodblocks became more and more intense and a high-temperature area appeared on the surface of the woodblocks. This area continuously expanded and was bright red (Fig 3d). Suddenly, a flame appeared at the high-temperature area (Fig 3e) and rapidly covered the upper surface of the woodblocks (Fig 3f).

### 3.2. Temperature variation process

**Fig 4:**
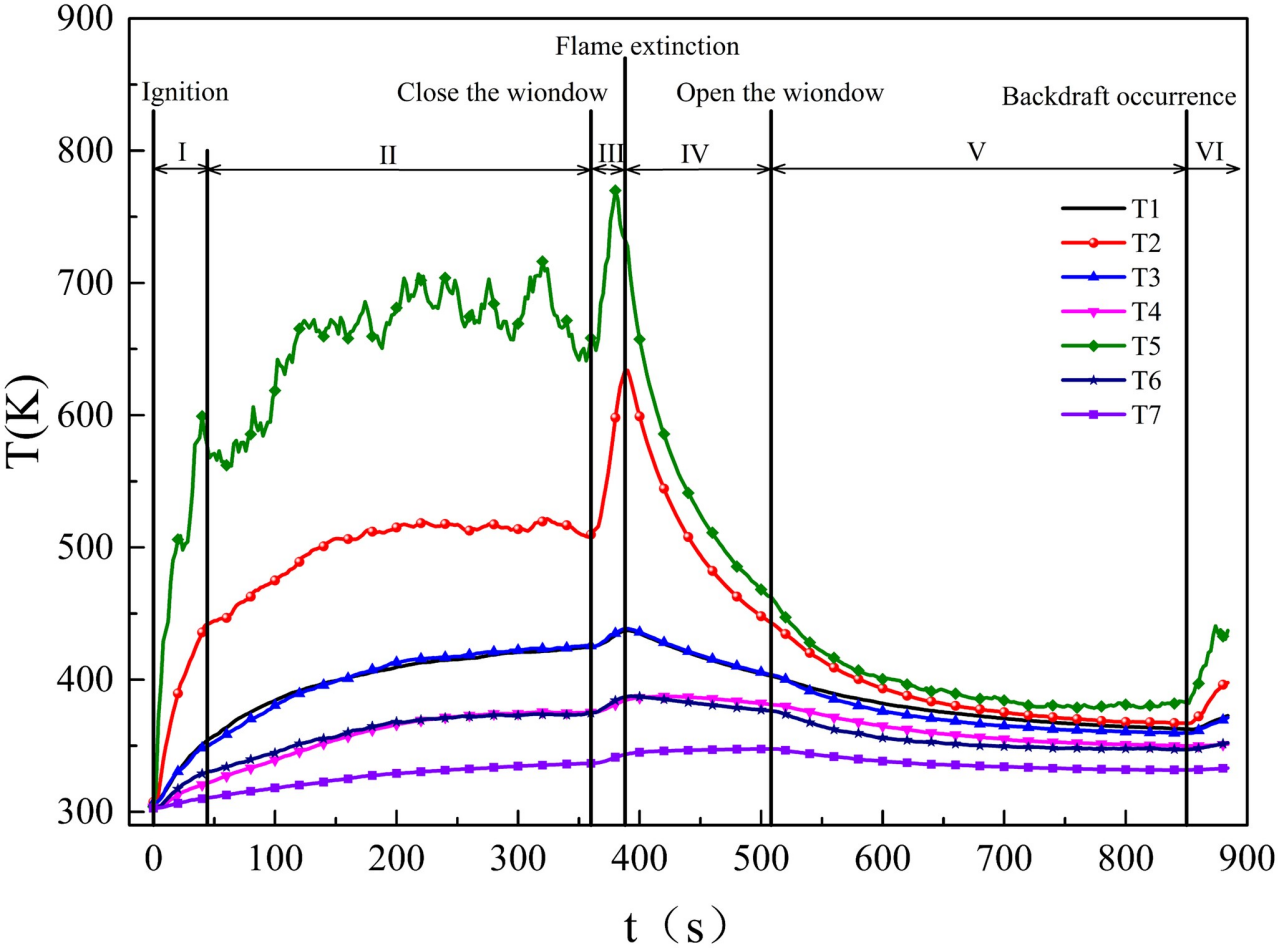
Temperature curves of the Test 8: (I) alcohol burning; (II) woodblocks steadily burning; (III) woodblocks transition from complete burning to incomplete burning; (IV) wood blocks smoldering; (V) gas exchange and smoldering intensely; (VI) wood blocks steadily burning again.

Fig 4 shows the temperature variation curves for Test 8. These curves include one descent stage, two stable stages, and three ascend stages. Every temperature curve has the same variation tendency, but the temperature values are quite different.

At the same time as ignition, the alcohol sprayed on the upper surface of the woodblocks started burning, and rapidly heated the surface of the woodblocks to the ignition temperature. Finally, when the alcohol exhausted, the woodblocks then burned steadily. Therefore, first, the temperature curves first rise rapidly, and then they gradually transmit stability. The shape of the temperature curve, recorded by the T5, was an irregular zigzag, because T5 was directly above the woodblocks, therefore, the flame swing influenced the temperature curve recorded by this thermocouple. The peak temperature curve appears when the flame is close to the thermocouple, and a trough appears when the flame is far from the thermocouple. Although T2 was also directly above the woodblocks, it was far from the top of the flame. The temperature curve recorded by this thermocouple is affected more by the heating airflow and less by the flame swing. Therefore, as compared with the temperature curve of T5, the temperature curve of T2 is less irregular, and the value is lower. The temperature curves recorded by the other thermocouples are smooth, because these curve variation tendencies caused by the circulation of gases in the compartment.

After the compartment window was closed, only a little heat dissipated from the compartment wall to the ambit by conduction, but the burning woodblocks still release heat during this period. Therefore, the temperature curves show an upward trend. The flames were extinguished when the oxygen concentration in the compartment was below the flammability limits. Then, the woodblocks transition to smoldering, released less heat, but heat continuously transferred from the compartment wall to ambient through conduction. Therefore, the temperature in the compartment decreased rapidly. However, the decreasing trend of the temperature curves gradually slows down, because the temperature difference between the inside and outside of the compartment decreased.

When the compartment window was opened, the temperature curves continue a downward trend, due to convective heat transfer between hot gas and fresh air through the compartment window. However, with an increase of oxygen concentration increase, the woodblocks’ smoldering intensity and the heat release increased. The downward trend of the temperature curves slows down and finally remains stable. When flames appeared on the surface of the wood blocks, the temperature curves began to increase.

Fig 4 shows temperature points for the upper part, where T2 is highest, and T1 is higher than T3, and for the middle part, where T5 is higher than T6and T6 is almost equal to T4. Fig 1 shows that T2 and T5 are temperature points directly above the woodblocks, T1 and T3 are temperature points on both sides of T2, and T4 and T6 are temperature points on both sides of T5. Therefore, on the horizontal plane, the highest temperature of hot smoke appeared at the middle position. In addition, T1 is higher thanT4, T4 is high than T7, and T3 is higher than T6, which means the temperature of smoke is higher in the upper part than in the lower part on the vertical plane. The reason for this is that under the action of the thermal buoyancy effect, higher temperature gas accumulates at the top of the compartment, and then moves to the bottom of the compartment. However, because T5 is close to the woodblocks and entrainment, the temperature of the hot smoke decreases. Therefore, although the location of T5 was lower than T2, the temperature value of T5 was higher than T2.

### 3.3. Gas concentration variation process

**Fig 5:**
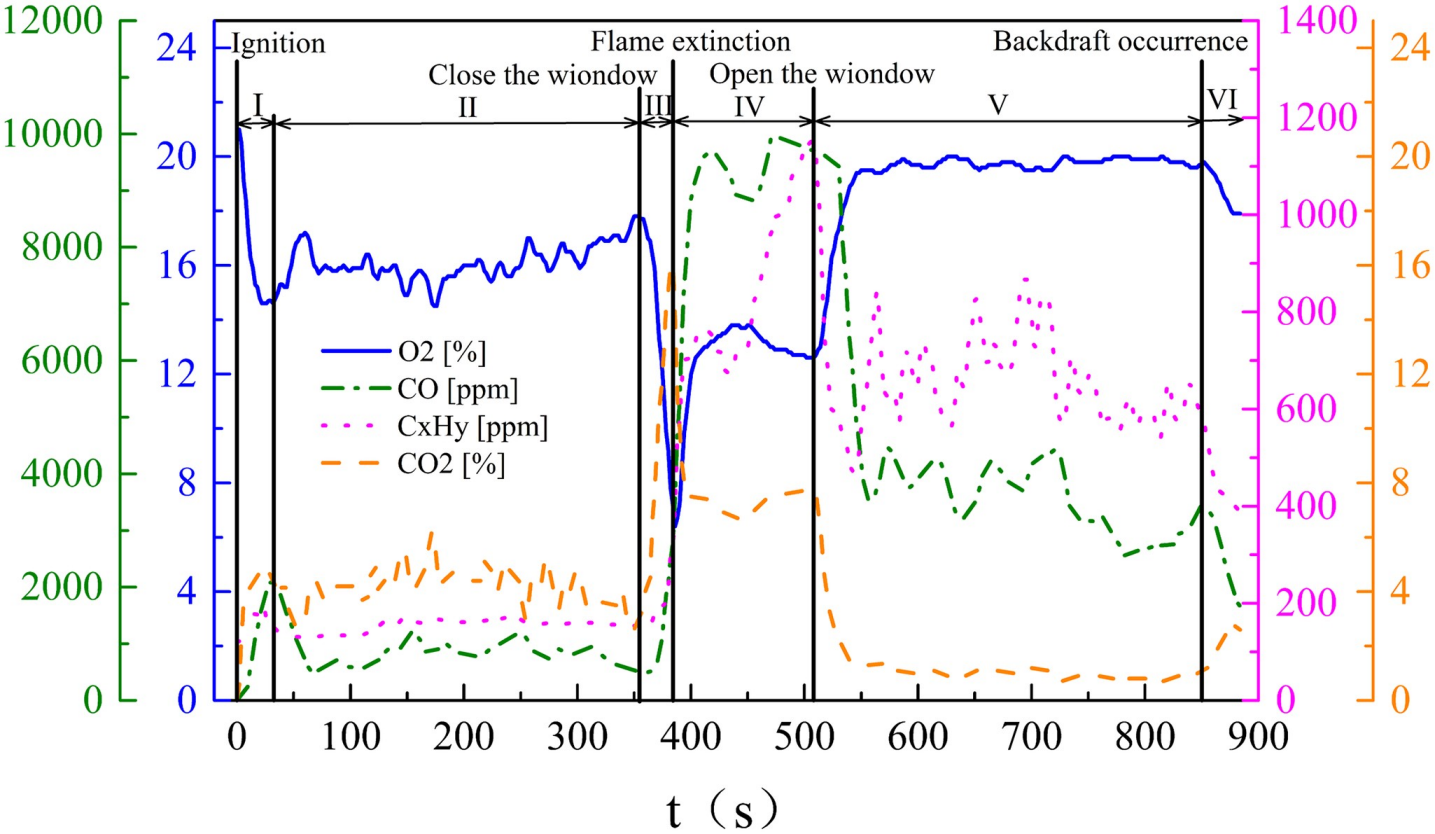
Gas component concentration curves of Test 8: (I) alcohol burning; (II) woodblocks steadily burning; (III) woodblocks transition from complete burning to incomplete burning; (IV) woodblocks smoldering; (V) gas exchange and intense smoldering; (VI) woodblocks steadily burning again.

During the experiments, the gas concentration is mainly affected by two factors: (1) the combustion form and intensity of the woodblocks and (2) the exchange of gas and fresh air. Fig 5 shows the gas concentration curves for Test 8. It shows that the values of the curves are quite different, and the change process shows stage characteristics. The changing trends of the O_2_ and CO_2_ concentrations are opposite and the changing trends of the CO and C_X_H_Y_ concentrations are similar. C_X_H_Y_ is hydrocarbon gas released by the woodblocks burning or smoldering, which is a product of the incomplete combustion of the woodblocks. In addition, the shape of the gas concentration curves is an irregular zigzag, due to the fact that gas sampling position was the fifith point, which was closest to the flame. Therefore, flame swing affects gas concentration.

At the same time as ignition, the alcohol sprayed on the upper surface of the woodblocks starts burning. Because burning consumes O_2_ and produces CO_2_, the O_2_ concentration rapidly decreases, meanwhile, the CO_2_ concentration increases. However, when the surface temperature of the woodblocks rises to the ignition temperature, the woodblocks also start burning. Then, the O_2_ and CO_2_ concentrations fluctuate and for Test 8, the corresponding values are 16% and 4%, respectively. During the above period, the compartment window was kept open, with good ventilation condition. There is no incomplete burning production produced, and therefore the CO and C_X_H_Y_ concentrations are low.

After the compartment window was closed, the O_2_ concentration in the compartment gradually decreases as the burning proceeds. At the same time, the CO_2_ concentration increases. Due to a decrease of O_2_ concentration, the combustion form of the woodblocks transition from complete burning to incomplete burning. Therefore, the CO and C_X_H_Y_ concentrations increase slightly. When the O_2_ concentration is finally below the flammability limits, the flame is extinguished, and the woodblocks transition to smoldering. Therefore, the O_2_ concentration slowly decreases, and the CO_2_ concentration gradually increases. But the CO and the C_X_H_Y_ concentrations rapidly increase, because a large number of incomplete products are generated during the smoldering process.

When the compartment window is opened, fresh air flows into the compartment and forms a gravity current. The O_2_ concentration rapidly increases, and the CO_2_ concentration rapidly decreases. Finally, these concentrations fluctuate near a value, but the woodblocks continue smoldering. Therefore, the consumption of O_2_ and the production of CO_2_ were lower than that of woodblocks steadily burning. During this period, the O_2_ concentration is (20%), and the CO_2_ concentration is (1%). Because woodblocks continued to smolder before backdraft occurred, the concentrations of CO and C_X_H_Y_ continued to be high after the window opened again, and the concentrations of CO and C_X_H_Y_ rapidly decreased once backdraft occurred.

### 3.4. The backdraft time

**Fig 6:**
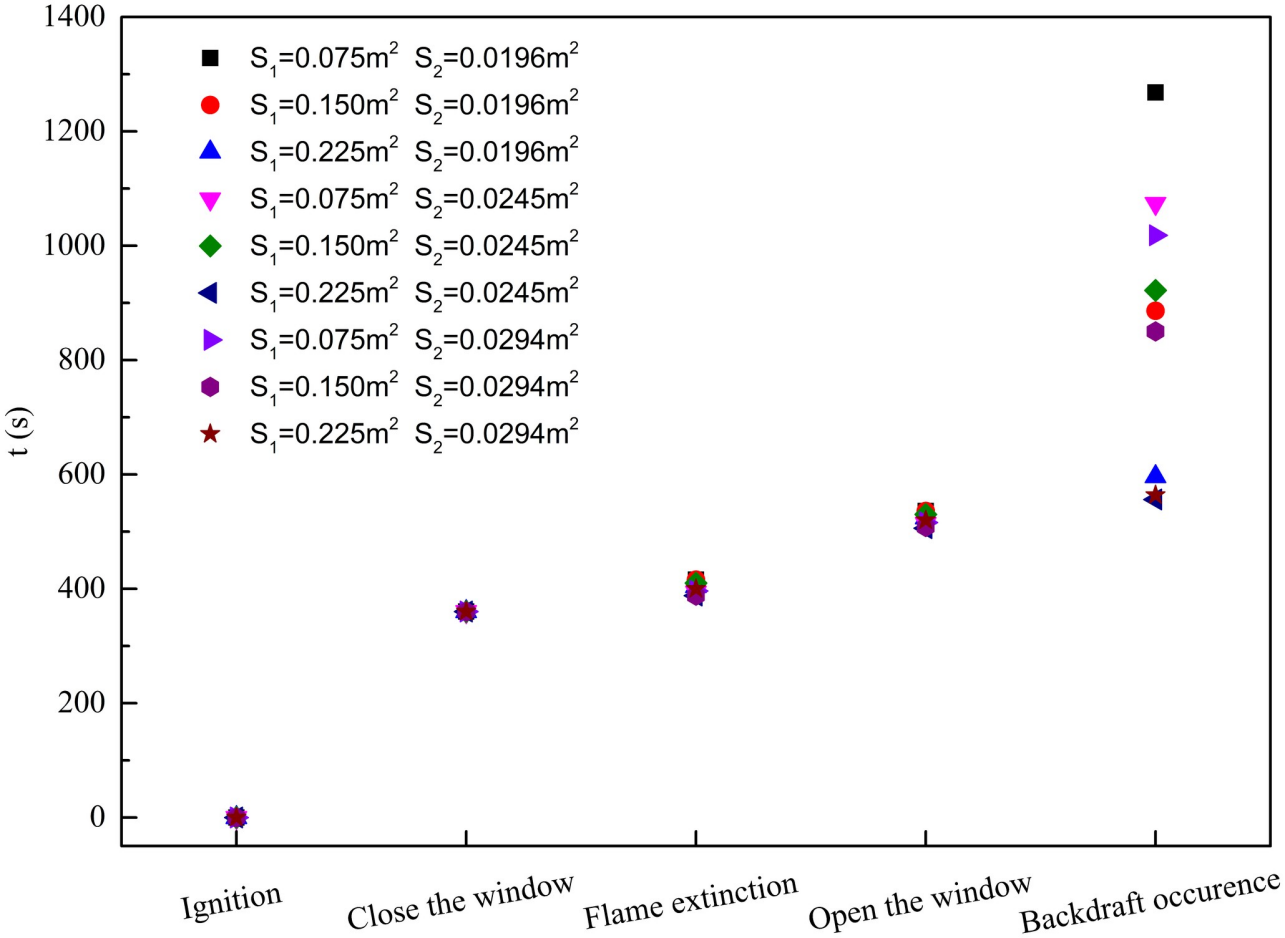
The corresponding times of the experimental processes.

**Fig 7:**
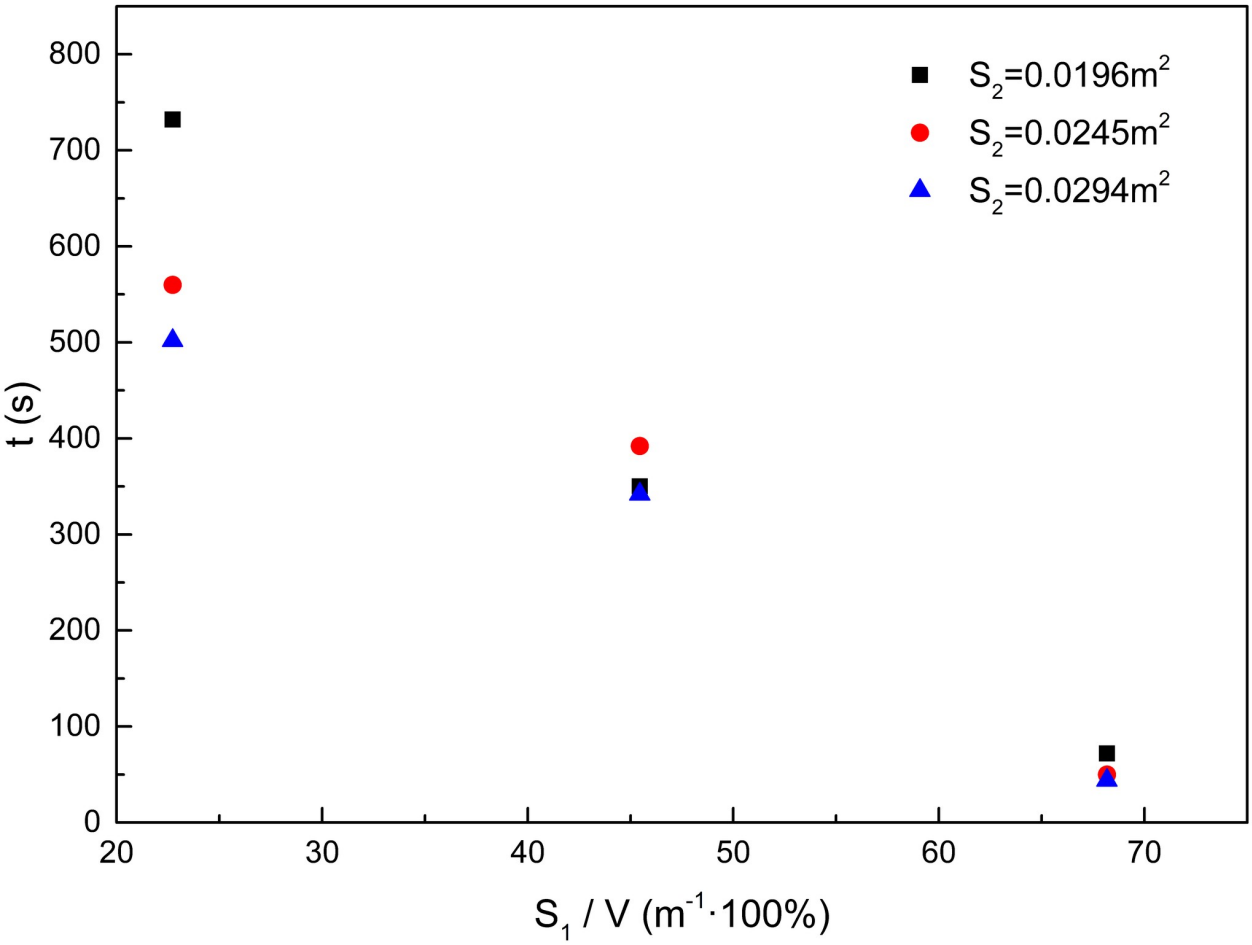
Effect of the compartment window area on backdraft time.

Fig 6 is the corresponding time of the experimental processes of each experiment. It can be observed that, although the experimental conditions are different, the corresponding times show regularity. The times that correspond to ignition and close the window are almost the same in different experiments, because they are all artificially controlled and not affected by the burning state. After the compartment window was closed, the flame extinguished because of hypoxia. This process is related to the burning state before the window closed, and therefore the required times have few differences but all less than one minute. From the time of flame extinction to opening the window, the time for all experiments was 120 seconds. After the compartment window opened again, the experimental conditions affected the backdraft time. The effects of the compartment window area and the upper surface area of the woodblocks on the backdraft time are shown in Fig 7 below.

Fig 7 suggests that when the upper surface areas of the woodblocks are equal, but the compartment window area is different, an increase in compartment window area would induces a decrease in backdraft time. The amounts of fresh air and smoke through the compartment window increase with an increase in the compartment window area. Thus, the combustible mixture concentration near the surface of the woodblocks rapidly changes. The flammability limits are achieved in a shorter time, and therefore the backdraft time is decreased.

**Fig 8:**
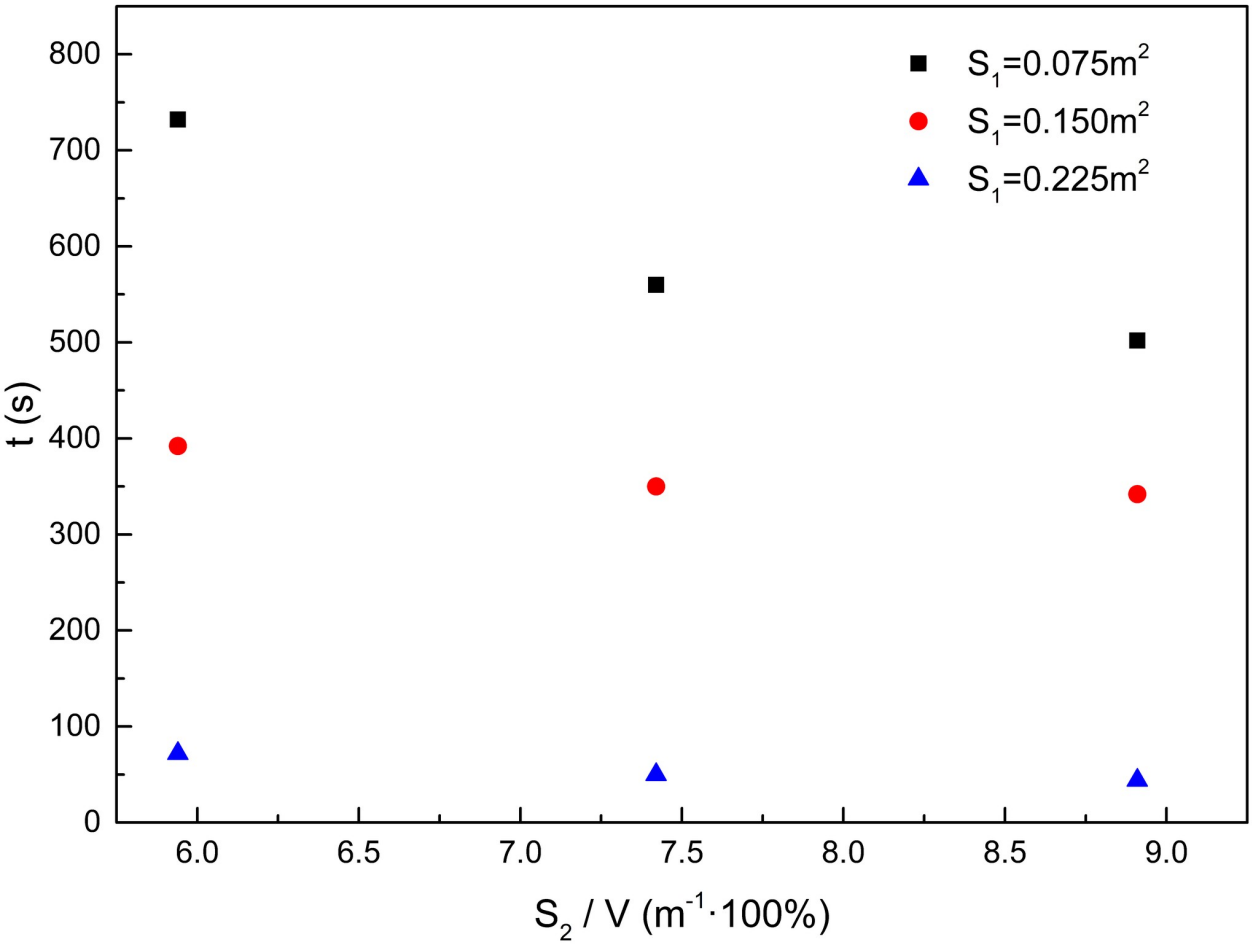
Effect of the upper surface area of woodblocks on backdraft time.

As shown in Fig 8, it suggests that when the compartment window area is equal but the upper surface areas of woodblocks are different, the backdraft time of the woodblocks decreases with an increase in the upper surface area of the woodblocks. After the compartment window opened again because the compartment window area is equal, the gas exchange situation inside and outside the compartment is similar. More heat is released from the woodblocks as the upper surface area of the woodblocks increases during the same time. The temperature of the woodblocks is higher, and the backdraft occurs in a short period of time.

When a fire breaks out in a ventilated confined space, the fuel surface area cannot be controlled; therefore, backdraft cannot be delayed by controlling the fuel surface area. However, backdraft can be delayed by controlling the ventilation vents of confined spaces. The positions of the vents should serve two functions. First, they allow high-temperature gas to flow out of the compartment to reduce the temperature and the combustible concentration. Second, they prevent fresh air flow into the compartment to prevent oxygen from reaching flammability limits. Therefore, we should open the upper vents of a ventilated confined space to avoid backdraft occurrence.

## 4. Conclusions

This paper investigated the backdraft phenomenon of solid fuel through small-scale experiments. The main conclusions are as follows:

1. Smoldering is a unique characteristic of the backdraft phenomenon of solid fuel. When compartment ventilation is improved, the smoldering of solid fuel gradually intensifies, and then backdraft would occur after a period of time.
2. The flammability limits that determined backdraft occurrence can be reached more easily when oxygen concentration and temperature are higher. For a large compartment window area, when more fresh air flows into the compartment, the oxygen concentration in the compartment increases. Woodblock combustion becomes more intensity with the increase of woodblock area, and the temperature in the compartment rise. Therefore, the backdraft time of solid fuel decreases with an increase in the compartment area and woodblock area.
3. A strategy for avoiding backdraft is suggested. When fighting a fire that breaks out in a ventilated confined compartment, an upper window in the compartment should be opened. Then, hot gas flows out, while the temperature and the combustible concentration decrease. Meanwhile, fresh air cannot flow in because of the hot gas expansion. This ventilation strategy could prevent oxygen reaching the flammability limit and avoid backdraft occurrences.
